# Bilayer Intelligent Omni-Surface-Assisted Full-Duplex Systems: Simultaneous Self-Interference Cancellation and Sum Rate Maximization

**DOI:** 10.3390/s24144535

**Published:** 2024-07-13

**Authors:** Yunxia Zhou, Qiucen Wu, Yu Zhu

**Affiliations:** School of Information Science and Technology, Fudan University, Shanghai 200433, China; yunxiazhou21@m.fudan.edu.cn (Y.Z.); qcwu21@m.fudan.edu.cn (Q.W.)

**Keywords:** bilayer intelligent omni-surface, full duplex, beamforming, self-interference cancellation, sum-rate maximization

## Abstract

Recently, reconfigurable intelligent surfaces (RISs) have attracted increasing attentions in the design of full-duplex (FD) systems due to their novel capability of propagation environment reconfiguration. However, in conventional RIS-assisted FD systems, the beamforming for self-interference cancellation (SIC) and sum rate maximization (SRM) are highly coupled during RIS optimization, which significantly degrades the system performance. To tackle this issue, we exploit a novel bilayer intelligent omni-surface (BIOS) structure in FD systems. Compared with the conventional RIS designs, the BIOS provides independent beams on both sides, thus enabling more flexible achievement of SRM and SIC. For the BIOS-assisted FD system, we first formulate an optimization problem to achieve SRM and efficient SIC simultaneously. Then, we exploit the relationship between the SRM and mean square error (MSE), and propose a weighted MSE minimization with SIC algorithm to solve the problem. Specifically, we jointly design the beamforming at the base station and the BIOS with manifold optimization while guaranteeing an SIC constraint. Furthermore, we theoretically derive a lower band for the BIOS size required for efficient SIC in FD systems. Simulation results indicate that the BIOS outperforms the conventional RIS designs in FD systems, and verify the accuracy of the derived lower bound for the BIOS size.

## 1. Introduction

With the explosive growth of wireless communication devices, the scarcity of available spectrum resources has become increasingly acute. Consequently, effectively utilizing existing spectrum resources has emerged as a pivotal factor in advancing wireless communication technology [1,2,3]. Recently, in-band full-duplex (FD) systems have drawn notable interest in the design of next-generation wireless communication systems due to their capability of transmitting and receiving data on the same frequency band concurrently. Compared to traditional half-duplex (HD) systems, such as time division duplex or frequency division duplex, FD systems can significantly enhance spectrum utilization and reduce the time delay of wireless communication systems [4,5]. Additionally, FD systems can significantly improve confidentiality while maintaining the same data rate [4]. Therefore, FD technology is critical for communication in ultra-dense network environments in the future.

Despite the advantages mentioned above, a prominent problem in FD systems is the inevitable self-interference (SI) emanating from the transmitter to the receiver, as its high intensity significantly interferes with uplink (UL) signals. To tackle this issue, three typical categories of SI cancellation (SIC) methods are discussed in the literature: transmission-domain processing, analog-domain processing, and digital-domain signal processing [4]. The transmission-domain SIC scheme involves suppressing SI through hardware design, such as physical isolation, shielding materials, and antennas with different polarization directions, thereby minimizing the power of SI signals before they reach the receiver [6,7,8]. The analog-domain SIC scheme focuses on suppressing SI in the radio frequency (RF) chain by adding isolators or designing circuits to generate signals that counteract the SI signals [9,10,11,12,13]. In [9], the authors added an additional RF chain at the transmitter, connected to the receiver. They estimated the SI channel and used the results to digitally preprocess the transmitted signal, generating a counteracting signal with the same amplitude but opposite phase to the SI, thus canceling it at the receiver. Similarly, in [10], an active RF chain was utilized to generate a signal compensating for the inevitable leakage from passive analog SIC. The digital-domain SIC scheme employs digital signal processing techniques on baseband signals to cancel residual SI, exploiting the spatial degrees of freedom provided by multi-antenna systems. Typically, digital SIC methods involve beamforming techniques such as zero-forcing (ZF), minimum mean square error (MMSE), and least squares [14,15,16,17]. In [14], the authors used ZF precoding at the transmitter and maximum ratio combining at the receiver to effectively cancel SI. In [15], the authors employed the MMSE algorithm to jointly design transmitting and receiving beamforming, aiming to minimize the mean square error (MSE) and reduce the SI. However, despite numerous attempts, these SIC methods come with high hardware expenses, substantial complexity, and constrained performance. Thus, the reconfigurable intelligent surface (RIS) is considered a promising technology and has attracted increasing attention in the design of FD systems. Specifically, RIS is a two-dimensional (2D) metasurface consisting of programmable, low-cost, and passive elements [18,19]. Due to its flexibility and low-cost nature, RIS has been extensively applied in wireless communication systems [20,21,22,23,24].

For the FD systems, RIS has been proven a promising technology to tackle the SIC and thus is widely investigated in the literature [25,26,27,28,29]. In [26], intelligent reflecting surface (IRS), as the most famous type of RIS, was exploited in FD systems to achieve efficient SIC. Specifically, the IRS smartly reconfigured the cascade signal emitted from the base station (BS) transmitter (${\mathrm{BS}}_{\mathrm{Tx}}$) to the IRS and then reflected to the BS receiver (${\mathrm{BS}}_{\mathrm{Rx}}$) to create destructive interference with the SI signal. Moreover, taking into account the quantization noise of analog-to-digital converters (ADCs), the authors of [27] considered a more practical scenario and proposed to jointly design the downlink (DL) precoding matrix and the IRS coefficients. However, due to hardware limitations, the IRS can only reflect the signal back to the same side [30]. Therefore, it can only service user equipment (UE) on the same side of the BS. To tackle this constraint, the authors of [28] first introduced intelligent omni-surface (IOS) to FD systems, which is named simultaneously transmitting and reflecting reconfigurable intelligent surface (STAR-RIS) in some studies [31]. Compared with the IRS, the IOS can simultaneously reflect and refract the incident signal to both sides [30,31]. Thus, it can extend the wireless coverage to the full space while suppressing the SI signal in FD systems.

Although previous studies have focused on the performance of RIS-assisted FD systems, there remains a notable gap in the literature regarding the simultaneous SIC and sum rate maximization (SRM) of DL and UL. Ref. [28] only addressed SI constraints targeting DL rate optimization, while [25] assumed perfect SIC when maximizing the sum rate (SR). However, minimizing the residual SI is crucial for effective air interface SIC as excessive SI will lead to the saturation of ADCs, preventing it from functioning properly [7]. Thus, it is necessary to investigate beamforming optimization for the SRM under practical SI constraints in RIS-assisted FD systems. Moreover, it is notable that although current IOS models offer both reflection and refraction capabilities, they cannot independently adjust reflecting and refracting phase shifts [30,31,32]. This limitation results in coupled beamforming on both sides, which leads to a serious performance loss in the FD system as the IOS is expected to simultaneously suppress the SI signal and enhance the system data rate.

To deal with these issues, in this paper, we introduce a novel bilayer intelligent omni-surface (BIOS) structure to simultaneously suppress the SI and enhance the system SR in FD systems. This BIOS structure deploys two neighboring IOS layers, and thus can ensure independent beamforming design on both sides [33]. Unlike [33], which utilizes both reflected and refracted beams for communication, our design employs the reflected signals for SIC and the refracted signal for communication, thus achieving both objectives simultaneously. Our approach is grounded in a practical scenario that considers the power constraint of the SI signal. The main contributions are summarized as follows:

- We introduce a novel BIOS in a multi-user FD system. With the unique bilayer structure, the BIOS can provide flexible beamforming on both sides and thus can simultaneously improve the SR and cancel the SI more effectively than the conventional RIS designs.
- For the BIOS-assisted multi-user FD system, we jointly optimize the beamforming at the BS and BIOS to maximize the SR of UL and DL. We ensure effective over-the-air SIC by constraining the residual SI power below a threshold, marking a distinct approach from that of other studies. To solve this problem, we establish the proof of equivalence between the SRM and a weighted MSE minimization (WMMSE) and then propose a novel SIC-WMMSE algorithm.
- To further guide the practical deployment of BIOS-assisted FD systems, we theoretically derive a lower bound of the BIOS size required for efficient SIC, which is proven to be quite close to the accurate size required in practical systems.
- We provide simulation results to show that the BIOS-assisted FD system significantly outperforms that of the conventional IOS due to its capability of independent beamforming on both sides.

Notations: Matrices and vectors are denoted by boldface upper case and boldface lower case, respectively. ${\left(\cdot \right)}^{T}$, ${\left(\cdot \right)}^{H}$, ${\left(\cdot \right)}^{-1}$, and ${\left(\cdot \right)}^{*}$, respectively, denote the transpose, Hermitian transpose, inverse, and conjugate. ${\left[\cdot \right]}_{ij}$ and ${\left[\cdot \right]}_{i}$ denote the (i,j)-th element of a matrix and the *i*-th element of a vector. $C^{M\times N}$ denotes the space of M×N complex-valued matrices. The trace, $l_{1}$ norm, and Frobenius norm are denoted by Tr(·), ${\left|\cdot \right|}_{1}$, and ${∥\cdot ∥}_{F}$, respectively. R{·} and E(·), respectively, denote the real-part and expectation operation. ∘ denotes Hadamard products. Furthermore, $I_{N}$ denotes the N×N identity matrix. diag(a) is a diagonal matrix with the elements of a on its main diagonal and diag(A) represents the extraction of the diagonal of A. CN(0,K) denotes the circularly symmetric complex Gaussian distribution with zero mean and covariance matrix K.

## 2. System Model and Problem Formulation

### 2.1. BIOS Structure

As mentioned in Section 1, hardware limitations prevent IOS from independently controlling the phases of refracted and reflected signals, resulting in symmetric beams on both sides. However, in the proposed FD system, we aim to utilize the reflected signals to cancel SI and for the refracted signals to communicate. Thus, the symmetric beams will degrade the system performance. Specifically, the cascaded reflected signal, which is emitted from the ${\mathrm{BS}}_{\mathrm{Tx}}$ into the IOS and then reflected to the ${\mathrm{BS}}_{\mathrm{Rx}}$, serves exclusively for SIC while the refracted signals aim to maximize SR. Moreover, refraction beamforming must align DL signals with the DL UEs and enhance UL reception, complicating the optimization problem due to increased coupling and non-convexity. Consequently, performance trade-offs are necessary to find an optimal balance.

**Figure 1:**
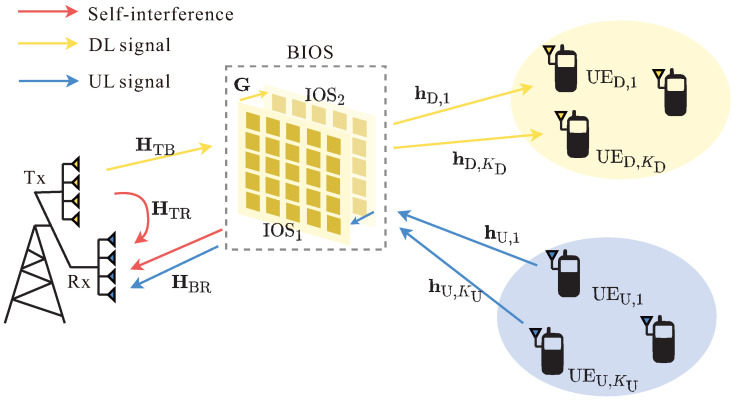
System model of the BIOS-assisted FD system.

To mitigate the constraints, we propose a BIOS-assisted FD system as shown in Figure 1, which can achieve simultaneous SIC and SRM by flexibly controlling the reflection and refraction beams on both sides of the BIOS [33,34]. The BIOS consists of two neighboring IOSs (referred to as ${\mathrm{IOS}}_{1}$ and ${\mathrm{IOS}}_{2}$). Situated proximately to the BS, ${\mathrm{IOS}}_{1}$ functions like a traditional IOS, managing both reflection and refraction, while ${\mathrm{IOS}}_{2}$ is set to the full-transmitting mode. With this arrangement, the reflected cascaded signals can be smartly designed by controlling the ${\mathrm{IOS}}_{1}$ to suppress SI signals. Furthermore, the SR can be maximized by independently controlling the reflection and refraction beams on both sides of the BIOS. Thus, the BIOS can achieve the dual-function target of SIC and SRM in FD systems better than the conventional RIS designs. Specifically, compared to traditional IRS, BIOS allows the BS to achieve greater service coverage by reflecting and refracting signals into the full space. Moreover, the capability of independent beamforming on both sides enables separate and effective management for SIC and SRM. Compared to single-layer IOS, the addition of ${\mathrm{IOS}}_{2}$ enables independent beams on both sides, allowing for more flexible adjustment. Additionally, compared to multilayer refractive RIS investigated in the previous studies [35,36], the BIOS is distinguished by its specific dual function of simultaneous and decoupled reflection and refraction, which enhances its performance in FD systems.

Without loss of generality, we assume that both ${\mathrm{IOS}}_{1}$ and ${\mathrm{IOS}}_{2}$ are uniform square planar arrays consisting of $M=M_{y}\times M_{z}$ elements, where $M_{y}=M_{z}$. Thus, the effective reflecting and refracting coefficient matrices of BIOS can be respectively presented as follows:

(1) $${\hat{\Phi }}_{r}=\sqrt{\xi_{r}}\Phi_{1},\;{\hat{\Phi }}_{t}=\sqrt{\epsilon_{t}\xi_{t}}\Phi_{2}G\Phi_{1},$$

where $\Phi_{1}\in C^{M\times M}$ and $\Phi_{2}\in C^{M\times M}$ denote the diagonal coefficient matrices of ${\mathrm{IOS}}_{1}$ and ${\mathrm{IOS}}_{2}$. $\xi_{r}$ and $\xi_{t}$ are the respective fractions of reflection and refraction signal power relative to the total power of the incoming signal for ${\mathrm{IOS}}_{1}$, satisfying the power constraint $\xi_{r}+\xi_{t}=1$. (Following the assumption in [32], we presume negligible penetration loss for IOS and BIOS. Nevertheless, the penetration loss will be an interesting and important issue that deserves further investigation.) Similarly, $\epsilon_{r}$ and $\epsilon_{t}$ denote those for ${\mathrm{IOS}}_{2}$ with $\epsilon_{r}+\epsilon_{t}=1$. According to the setup, $\epsilon_{r}=0$ and $\epsilon_{t}=1$. $G\in C^{M\times M}$ denotes the channel between ${\mathrm{IOS}}_{1}$ and ${\mathrm{IOS}}_{2}$.

### 2.2. System Model

Consider a narrowband multi-input single-output (MISO) FD system comprising a BS, a BIOS, $K_{D}$ DL UEs, and $K_{U}$ UL UEs in Figure 1. We assume that all the UEs are single-antenna, ${\mathrm{BS}}_{\mathrm{Tx}}$ employs $N_{T}$ transmit antennas, and ${\mathrm{BS}}_{\mathrm{Rx}}$ has $N_{R}$ receive antennas. The BIOS is deployed significantly close to the BS to ensure the reflected signals are strong enough to efficiently suppress the SI signals, so we assume that there is no line-of-sight (LoS) path between the BS and UEs [28].

For this FD system, the DL signal from the BS is denoted as $s_{D}=Fx_{D}$ with $E\{|s_{D}{|}^{2}\}=P_{s}$. Similarly, $F=\left[f_{1},\cdots ,f_{K_{D}}\right]\in C^{N_{T}\times K_{D}}$ denotes the precoder at the BS, $x_{D}=\left[x_{D,1},\cdots ,\right.{\left.x_{D,K_{D}}\right]}^{T}\in C^{K_{D}\times 1}$ denotes the original symbol vector, and $x_{U,l}$ is the symbol transmitted by the *l*-th UL UE with the power constraint $E\{|x_{U,l}{|}^{2}\}=P_{U}$. The channels between the ${\mathrm{BS}}_{\mathrm{Tx}}$-${\mathrm{IOS}}_{1}$, ${\mathrm{BS}}_{\mathrm{Tx}}$-${\mathrm{BS}}_{\mathrm{Rx}}$, ${\mathrm{BS}}_{\mathrm{Rx}}$-${\mathrm{IOS}}_{1}$, ${\mathrm{IOS}}_{2}$-${\mathrm{UE}}_{D,k}$, and ${\mathrm{IOS}}_{2}$-${\mathrm{UE}}_{U,l}$ are denoted, respectively, as $H_{\mathrm{TB}}\in C^{M\times N_{T}}$, $H_{\mathrm{TR}}\in C^{N_{R}\times N_{T}}$, $H_{\mathrm{BR}}\in C^{M\times N_{R}}$, $h_{D,k}\in C^{M\times 1}$, and $h_{U,l}\in C^{M\times 1}$. Therefore, the DL received signal at the *k*-th DL UE and the UL received signal at the ${\mathrm{BS}}_{\mathrm{Rx}}$ can be respectively represented as follows:   

(2) $$y_{D,k}=\sqrt{\xi_{t}}h_{D,k}^{H}\Phi_{2}G\Phi_{1}H_{\mathrm{TB}}f_{k}x_{D,k}+\underset{n_{D,k}^{\mathrm{MU}}}{\underset{︸}{\sum_{i=1,i\neq k}^{K_{D}}\sqrt{\xi_{t}}h_{D,k}^{H}\Phi_{2}G\Phi_{1}H_{\mathrm{TB}}f_{i}x_{D,i}}}+n_{D,k},$$

(3) $$\begin{array}{cc} y_{U} & =\sqrt{\xi_{t}}H_{\mathrm{BR}}^{H}\Phi_{1}G^{H}\Phi_{2}h_{U,l}x_{U,l}+\underset{n_{U,l}^{\mathrm{MU}}}{\underset{︸}{\sum_{i=1,i\neq l}^{K_{U}}\sqrt{\xi_{t}}H_{\mathrm{BR}}^{H}\Phi_{1}G^{H}\Phi_{2}h_{U,i}x_{U,i}}}\\ \; & +\underset{n^{\mathrm{SI}}}{\underset{︸}{\left(H_{\mathrm{TR}}+\sqrt{\xi_{r}}H_{\mathrm{BR}}^{H}\Phi_{1}H_{\mathrm{TB}}\right)\sum_{i=1}^{K_{D}}f_{i}x_{D,i}}}+n_{U}, \end{array}$$

where $n_{D,k}^{\mathrm{MU}}$ represents the interference from other DL UEs for the *k*-th DL UE and $n_{U,l}^{\mathrm{MU}}$ represents the interference from other UL UEs for the *l*-th UL UE. $n^{\mathrm{SI}}$ is the SI at the BS. $n_{D,k}$ denotes the Gaussian noise at the *k*-th DL UE with mean zero and variance $\sigma_{D}^{2}$, and $n_{U}$ denotes the noise at the BS satisfying the distribution of $n_{U}∼\mathrm{CN}\left(0,\sigma_{U}^{2}I\right)$.

### 2.3. Channel Model

Before problem formulation, we first present detailed channel modeling. As the BIOS is deployed at the BS to achieve efficient SIC, $H_{\mathrm{TB}}$, $H_{\mathrm{TR}}$, and  $H_{\mathrm{BR}}$ are considered near-field channels without non-line-of-sight (NLoS) paths. Thus, considering the properties of spherical waves, we model the LoS path gain from the *n*-th receive antenna of ${\mathrm{BS}}_{\mathrm{Tx}}$ to the *m*-th element of ${\mathrm{IOS}}_{1}$ as follows [37]:

(4) $${\left[H_{\mathrm{TB}}\right]}_{m,n}=\sqrt{\frac{a^{2}G_{\mathrm{TB}}F_{\mathrm{TB}}\left(\theta_{m,n}^{r},\phi_{m,n}^{r}\right)}{4\pi d_{m,n}^{2}}}\exp\left(\frac{-j2\pi d_{m,n}}{\lambda }\right),$$

where *a* is the size of scattering elements, λ is the carrier wavelength, and $d_{m,n}$ is the distance between the *n*-th antenna of ${\mathrm{BS}}_{\mathrm{Tx}}$ and the *m*-th element of ${\mathrm{IOS}}_{1}$. $\theta_{m,n}^{r}$ and $\phi_{m,n}^{r}$, respectively, denote the elevation and azimuth angle of the received signal at the *m*-th element of ${\mathrm{IOS}}_{1}$. Moreover, $G_{\mathrm{TB}}=2$ and $F_{\mathrm{TB}}\left(\theta ,\phi \right)=\left|\cos\theta \right|$, respectively, denote parameters related to the equivalent antenna gain and the normalized power radiation pattern between ${\mathrm{BS}}_{\mathrm{Tx}}$ and ${\mathrm{IOS}}_{1}$.

Similarly, the channel gain of $H_{\mathrm{BR}}$, $H_{\mathrm{TR}}$, and G can be modeled as follows:

(5) $${\left[H_{\mathrm{BR}}\right]}_{m,n}=\sqrt{\frac{a^{2}G_{\mathrm{BR}}F_{\mathrm{BR}}\left(\theta_{m,n}^{r},\phi_{m,n}^{r}\right)}{4\pi d_{m,n}^{2}}}\exp\left(\frac{-j2\pi d_{m,n}}{\lambda }\right),$$

(6) $${\left[H_{\mathrm{TR}}\right]}_{m,n}=\sqrt{\frac{a^{2}}{4\pi d_{m,n}^{2}}}\exp\left(\frac{-j2\pi d_{m,n}}{\lambda }\right),$$

(7) $${\left[G\right]}_{m,n}=\sqrt{\frac{a^{2}G_{\mathrm{II}}F_{\mathrm{II}}\left(\theta_{m,n}^{r},\phi_{m,n}^{r}\right)}{4\pi d_{m,n}^{2}}}\exp\left(\frac{-j2\pi d_{m,n}}{\lambda }\right),$$

where $G_{\mathrm{BR}}=2$, $G_{\mathrm{II}}=1$, $F_{\mathrm{BR}}\left(\theta ,\phi \right)=\left|\cos\theta \right|$, and $F_{\mathrm{II}}\left(\theta ,\phi \right)={\left|\cos\theta \right|}^{2}$.

Considering the NLoS paths, $h_{D,k}$ and $h_{U,l}$ can be modeled as Rician channels. Specifically, the channel gain between the *k*-th DL UE and *m*-th element of ${\mathrm{IOS}}_{2}$ can be modeled as follows:

(8) $${\left[h_{D,k}\right]}_{m}=\sqrt{\frac{\kappa }{1+\kappa }}h_{m}^{\mathrm{LoS}}+\sqrt{\frac{1}{1+\kappa }}h_{m}^{\mathrm{NLoS}},$$

(9) $$h_{m}^{\mathrm{LoS}}=\sqrt{\alpha_{0}{\left(\frac{d_{m}}{d_{0}}\right)}^{-\beta }G_{\mathrm{UE}}F_{\mathrm{UE}}\left(\theta_{m}^{t},\phi_{m}^{t}\right)}\exp\left(\frac{-j2\pi d_{m}}{\lambda }\right),$$

(10) $$h_{m}^{\mathrm{NLoS}}=\sqrt{\alpha_{0}{\left(\frac{d_{m}}{d_{0}}\right)}^{-\beta }G_{\mathrm{UE}}F_{\mathrm{UE}}\left(\theta_{m}^{t},\phi_{m}^{t}\right)}h_{m}^{s},$$

where κ is the Rician factor. $h_{m}^{\mathrm{LoS}}$ and $h_{m}^{\mathrm{NLoS}}$, respectively, denote the LoS and NLoS path gain from the *m*-th element of ${\mathrm{IOS}}_{2}$ to the *k*-th DL UE. $d_{m}$ denotes the distance between the *m*-th element of ${\mathrm{IOS}}_{2}$ and the *k*-th DL UE. $\alpha_{0}$ denotes the pass loss of the reference distance $d_{0}$ with the path loss exponent β. $h_{m_{1},n}^{s}∼\mathrm{CN}\left(0,1\right)$ denotes the cumulative effect of a large number of scattered paths. Moreover, $G_{\mathrm{UE}}=2$, $F_{\mathrm{UE}}\left(\theta ,\phi \right)=\left|\cos\theta \right|$. Similarly, $h_{U,l}$ can be modeled in the same way.

### 2.4. Problem Formulation

According to (2) and (3), the data rate of the *k*-th DL UE can be expressed as

(11) $$R_{D,k}=\log_{2}\left(1+\frac{{\left|\sqrt{\xi_{t}}h_{D,k}^{H}\Phi_{2}G\Phi_{1}H_{\mathrm{TB}}f_{k}\right|}^{2}}{\sum_{\begin{array}{c} i=1,i\neq k \end{array}}^{K_{D}}{\left|\sqrt{\xi_{t}}h_{D,k}^{H}\Phi_{2}G\Phi_{1}H_{\mathrm{TB}}f_{i}\right|}^{2}+\sigma_{D}^{2}}\right).$$

Similarly, the UL data rate of the *l*-th UL UE is given by

(12) $$R_{U,l}=\log_{2}\left|I+\frac{P_{U}\hat{H}h_{U,l}h_{U,l}^{H}{\hat{H}}^{H}}{\sum_{\begin{array}{c} j=1,j\neq l \end{array}}^{K_{U}}P_{U}\hat{H}h_{U,j}h_{U,j}^{H}{\hat{H}}^{H}+\sum_{i=1}^{K_{D}}H_{\mathrm{SI}}f_{i}f_{i}^{H}H_{\mathrm{SI}}^{H}+\sigma_{U}^{2}I}\right|,$$

where $\hat{H}=\sqrt{\xi_{t}}H_{\mathrm{BR}}^{H}\Phi_{1}G^{H}\Phi_{2}$, $H_{\mathrm{SI}}=H_{\mathrm{TR}}+\sqrt{\xi_{r}}H_{\mathrm{BR}}^{H}\Phi_{1}H_{\mathrm{TB}}$.

In this paper, we jointly optimize the beamforming design at the BS and BIOS to maximize the SR of UL and DL while effectively managing SIC. It is worth noting that excessive residual SI will cause the saturation of ADC, preventing it from functioning properly. However, most of the existing works are based on perfect SIC. To tackle this issue, we take into account the hardware limitations and then impose a constraint to maintain the residual SI power below a threshold, which is different from other papers. Specifically, the problem can be formulated as follows:

(13a) $$\begin{array}{cc} \max_{\Phi_{1},\Phi_{2},F}\; & \sum_{l=1}^{K_{U}}R_{U,l}+\sum_{k=1}^{K_{D}}R_{D,k} \end{array}$$

(13b) $$\begin{array}{cc} s.t.\;\; & {∥H_{\mathrm{SI}}F∥}_{F}^{2}/N_{R}\leq \gamma , \end{array}$$

(13c) $$\begin{array}{cc} \; & {\left|\Phi_{1}\right|}_{ii}={\left|\Phi_{2}\right|}_{jj}=1,\forall i,j, \end{array}$$

(13d) $$\begin{array}{cc} \; & \mathrm{Tr}\left(FF^{H}\right)\leq P_{s}. \end{array}$$

Constraint (13b) maintains the residual SI power below a threshold to achieve efficient SIC. Constraints (13c) and (13d) result from the constant modulus constraint of the BIOS scattering elements and the power limitation at the BS, respectively.

## 3. Beamforming Design for BIOS-FD Systems

Problem (13) is difficult to solve directly due to the severely coupled variables and non-convex constraints. Thus, we propose an SIC-WMMSE method to transform the primal problem into an equivalent weighted MSE minimization problem, and then employ alternating optimization (AO) to solve it.

### 3.1. SIC-WMMSE Approach

Due to the multiple coupled variables and non-convex constraints, problem (13) is difficult to solve directly. However, by leveraging the relationship between MSE and data rate, we convert the primal problem (13) into an equivalent WMMSE problem as follows:

(14) $$\begin{array}{cc} \min_{\begin{array}{c} \Phi_{1},\Phi_{2},F_{,}\\ a_{D,k},u_{D,k},a_{U,l},u_{U,l} \end{array}} & f=\sum_{k=1}^{K_{D}}\left(a_{D,k}e_{D,k}-\log_{2}\left|a_{D,k}\right|\right)+\sum_{l=1}^{K_{U}}\left(a_{U,l}e_{U,l}-\log_{2}\left|a_{U,l}\right|\right)\\ \;\;\;\;\;\;\;\;\;s.t.\;\; & {∥H_{\mathrm{SI}}F∥}_{F}^{2}/N_{R}\leq \gamma ,\\ \; & {\left|\Phi_{1}\right|}_{ii}={\left|\Phi_{2}\right|}_{jj}=1,\forall i,j,\\ \; & \mathrm{Tr}\left(FF^{H}\right)\leq P_{s}, \end{array}$$

where $a_{D,k}$ and $a_{U,l}$ are the weighting coefficients for the *k*-th DL UE and the *l*-th UL UE, respectively. $u_{D,k}^{*}$ is the receive coefficient at the *k*-th DL UE and $u_{U,l}^{H}$ is the receive combiner for the *l*-th UL UE at the ${\mathrm{BS}}_{\mathrm{Rx}}$, respectively. $e_{D,k}$ and $e_{U,l}$, respectively, denote the MSEs of the *k*-th DL UE and the *l*-th UL UE as follows:

(15) eD,k=EuD,k*yD,k−xD,k2=uD,k*h^D,kHfk−1uD,k*h^D,kHfk−1H+∑i=1i≠kKDuD,k*uD,kh^D,kHfifiHh^D,k+σD,k2uD,k*uD,k=uD,k*uD,kh^D,kHFFHh^D,k−2ReuD,k*h^D,kHfk+σD,k2uD,k*uD,k+1,

(16) eU,l=EuU,lHyU−xU,l2=PUuU,lHh^U,l−1uU,lHh^U,l−1H+∑i=1i≠lKUPUuU,lHh^U,ih^U,iHuU,l+uU,lHHSIFFHHSIHuU,l+σU2uU,lHIuU,l=uU,lHPUH^UH^UH+HSIFFHHSIH+σU2IuU,l−2PUReuU,lHl^U,l+1,

where ${\hat{h}}_{D,k}^{H}=\sqrt{\xi_{t}}h_{D,k}^{H}\Phi_{2}G\Phi_{1}H_{\mathrm{TB}}$ represents the equivalent channel of the *k*-th DL UE for simplification and ${\hat{H}}_{D}^{H}={\left[{\hat{h}}_{D,1},\cdots ,{\hat{h}}_{D,K_{D}}\right]}^{H}$. Similarly, ${\hat{h}}_{U,l}=\sqrt{\xi_{t}}H_{\mathrm{BR}}^{H}\Phi_{1}G^{H}\Phi_{2}h_{U,l}$ represents the equivalent channel of the *l*-th UL UE for simplification and ${\hat{H}}_{U}=\left[{\hat{h}}_{U,1},\cdots ,{\hat{h}}_{U,K_{U}}\right]$. The proof of the equivalence between problems (13) and (14) can be found in Appendix A. For a more concise representation, problem (14) can be rewritten as follows by transforming the scalars into matrices:

(17) $$\begin{array}{cc} \min_{\Phi_{1},\Phi_{2},F,U_{D},A_{D},U_{U},A_{U}} & f=\mathrm{Tr}\left(A_{D}E_{D}\right)-\log\det\left(A_{D}\right)+\mathrm{Tr}\left(A_{U}E_{U}\right)-\log\det\left(A_{U}\right)\\ \;\;\;\;\;\;\;\;\;s.t.\;\; & {∥H_{\mathrm{SI}}F∥}_{F}^{2}/N_{R}\leq \gamma ,\\ \; & {\left|\Phi_{1}\right|}_{ii}={\left|\Phi_{2}\right|}_{jj}=1,\forall i,j,\\ \; & \mathrm{Tr}\left(FF^{H}\right)\leq P_{s}, \end{array}$$

where $E_{D}=\mathrm{diag}\left(e_{D,1},\cdots ,e_{D,K_{D}}\right)$ and $E_{U}=\mathrm{diag}\left(e_{U,1},\cdots ,e_{U,K_{U}}\right)$, respectively, denote diagonal matrices containing the MSEs of each DL and UL UE. $A_{D}=\mathrm{diag}\left(a_{D,1},\cdots ,a_{D,K_{D}}\right)$ and $A_{U}=\mathrm{diag}\left(a_{U,1},\cdots ,a_{U,K_{U}}\right)$, respectively, denote diagonal matrices containing the weighting coefficients of each DL and UL UE. $U_{D}=\mathrm{diag}\left(u_{D,1},\cdots ,u_{D,K_{D}}\right)$ denotes the diagonal matrix containing the receive coefficients of each DL UE, and $U_{U}=\left[u_{U,1},\ldots ,u_{U,K_{U}}\right]$ denotes the matrix containing the receive combiners of each UL UE.

Although we have transformed the SRM problem into an equivalent WMMSE problem (17), it is still difficult to solve it directly due to the highly non-convex constraints. Thus, we propose an efficient way to deal with the residual SI power constraint (13b). Specifically, move the SI suppression term into the objective function with a penalty factor ρ, resulting in a new optimization problem as follows:

(18) $$\begin{array}{cc} \min_{\Phi_{1},\Phi_{2},F,U_{D},A_{D},U_{U},A_{U}} & f=\mathrm{Tr}\left(A_{D}E_{D}\right)-\log\det\left(A_{D}\right)+\mathrm{Tr}\left(A_{U}E_{U}\right)-\log\det\left(A_{U}\right)+\rho \zeta \\ \;\;\;\;\;\;\;\;\;s.t.\;\; & {\left|\Phi_{1}\right|}_{ii}={\left|\Phi_{2}\right|}_{jj}=1,\forall i,j,\\ \; & \mathrm{Tr}\left(FF^{H}\right)\leq P_{s}, \end{array}$$

where $\zeta =\frac{1}{N_{R}}{∥H_{\mathrm{SI}}F∥}_{F}^{2}$ is the power of the SI signal.

In order to facilitate the optimization of each variable, we substitute all variables into objective function (18) to derive a more detailed expression of *f*, which can be expressed as follows:

(19) $$f=f_{D}+f_{U}+f_{\mathrm{SI}},$$

where

(20) fD=TrADED−logdetAD=TrFHH^DADUDHUDH^DHF−2ReTrADUDHH^DHF+TrADσDUDHUD+TrAD−log2AD=TrξtFHHTBHΦ1HGHΦ2HHDADUDHUDHDHΦ2GΦ1HTBF+TrADσDUDHUD−2ReTrξtADUDHHDHΦ2GΦ1HTBF+TrAD−log2AD,

(21) fU=TrAUEU−logdetAU=TrUUAUUUHPUH^UH^UH+HSIFFHHSIH+σU2I−2RePUTrH^UAUUUH+TrAU−log2AU=TrUUAUUUHξtPUHBRHΦ1GHΦ2HUHUHΦ2HGΦ1HHBR+HTR+ξrHBRHΦ1HTBFFHHTR+ξrHRRHΦ1HTBH+σU2I−2ReTrPUξtUUAUHBRHΦ1GHΦ2HU+TrAU−log2AU,

(22) $$\begin{array}{cc} f_{\mathrm{SI}} & =\frac{\rho }{N_{R}}{∥H_{\mathrm{TR}}+\sqrt{\xi_{r}}H_{\mathrm{BR}}^{H}\Phi_{1}H_{\mathrm{TB}}∥}_{F}^{2}\\ \; & =\frac{\rho }{N_{R}}\mathrm{Tr}\left(\left(H_{\mathrm{TR}}+\sqrt{\xi_{r}}H_{\mathrm{BR}}^{H}\Phi_{1}H_{\mathrm{TB}}\right){\left(H_{\mathrm{TR}}+\sqrt{\xi_{r}}H_{\mathrm{BR}}^{H}\Phi_{1}H_{\mathrm{TB}}\right)}^{H}\right)\\ \; & =\frac{\rho }{N_{R}}\mathrm{Tr}\left(H_{\mathrm{TR}}H_{\mathrm{TR}}^{H}+\sqrt{\xi_{r}}H_{\mathrm{TR}}H_{\mathrm{TB}}^{H}\Phi_{1}^{H}H_{\mathrm{BR}}\right.\\ \; & \;\left.+\sqrt{\xi_{r}}H_{\mathrm{BR}}^{H}\Phi_{1}H_{\mathrm{TB}}H_{\mathrm{TR}}^{H}+\xi_{r}H_{\mathrm{BR}}^{H}\Phi_{1}H_{\mathrm{TB}}H_{\mathrm{TB}}^{H}\Phi_{1}^{H}H_{\mathrm{BR}}\right). \end{array}$$

### 3.2. Optimize $A_{D}$, $A_{U}$, $U_{D}$, $U_{U}$ with Fixed F, $\Phi_{1}$, $\Phi_{2}$

According to the WMMSE design approach [28] and the derivation in Appendix A, $A_{D}$, $A_{U}$, $U_{D}$, and $U_{U}$ all have closed-form solutions when other variables are fixed, which can be expressed as follows:

(23) aD,k★=eD,k−1=uD,k*uD,kh^D,kHFFHh^D,k−2ReuD,k*h^D,kHfk+σD,k2uD,k*uD,k+1−1,

(24) $${u_{D,k}}^{★}={\left({\hat{h}}_{D,k}^{H}FF^{H}{\hat{h}}_{D,k}+\sigma_{D,k}^{2}\right)}^{-1}{\hat{h}}_{D,k}^{H}f_{k},$$

(25) aU,l★=eU,l−1=uU,lHPUH^UH^UH+HSIFFHHSIH+σU2IuU,l−2PUReuU,lHh^U,l+1−1,

(26) $${u_{U,l}}^{★}=\sqrt{P_{U}}{\left(P_{U}{\hat{H}}_{U}{\hat{H}}_{U}^{H}+H_{\mathrm{SI}}FF^{H}H_{\mathrm{SI}}^{H}+\sigma_{U}^{2}I\right)}^{-1}{\hat{h}}_{U,l}.$$

### 3.3. Optimize F with Fixed $A_{D}$, $A_{U}$, $U_{D}$, $U_{U}$, $\Phi_{1}$, $\Phi_{2}$

When $A_{D}$, $A_{U}$, $U_{D}$, $U_{U}$, $\Phi_{1}$, and $\Phi_{2}$ are fixed, problem (18) is reduced into a sub-problem for the optimization of F with the power constraint, which can be rewritten as follows by ignoring constant terms:

(27) minFf(F)=TrUUAUUUHHSIFFHHSIH+TrFHH^DADUDHUDH^DHF−2ReTrADUDHH^DHF+ρNRTrHSIFFHHSIHs.t.TrFHF≤Ps.

It can be observed that sub-problem (27) is convex. Thus, with other parameters fixed, the optimal F can be obtained by utilizing the Karush–Kuhn–Tucker (KKT) conditions as

(28) $$F^{★}=\tilde{F}{\hat{H}}_{D}A_{D}U_{D},$$

where $\tilde{F}={\left(H_{\mathrm{SI}}^{H}U_{U}A_{U}U_{U}^{H}H_{\mathrm{SI}}+{\hat{H}}_{D}A_{D}U_{D}^{H}U_{D}{\hat{H}}_{D}^{H}+\frac{\rho }{N_{R}}H_{\mathrm{SI}}^{H}H_{\mathrm{SI}}+\delta I\right)}^{-1}$. δ is the KKT coefficient whose optimal solution can be obtained through a one-dimensional search based on the power constraint. The follow two subsections focus on the optimization of BIOS, i.e., $\Phi_{1}$, $\Phi_{2}$.

### 3.4. Optimize $\Phi_{1}$ with Fixed F, $\Phi_{2}$, $A_{D}$, $A_{U}$, $U_{D}$, $U_{U}$

In this subsection, we will optimize $\Phi_{1}$ by fixing other variables. By further defining $H_{D}=\left[h_{D,1},\cdots ,h_{D,K_{D}}\right]$, $H_{U}=\left[h_{U,1},\cdots ,{\hat{h}}_{U,K_{U}}\right]$, $\bar{H}=H_{U}^{H}\Phi_{2}^{H}G^{H}$, $\bar{F}=H_{\mathrm{TB}}F$, $H_{\mathrm{fle}}=H_{\mathrm{BR}}U_{U}A_{U}U_{U}^{H}H_{\mathrm{BR}}^{H}$, and $H_{\mathrm{fra}}=G^{H}\Phi_{2}^{H}H_{D}U_{D}A_{D}U_{D}^{H}H_{D}^{H}\Phi_{2}G$, the objective function in (18) related to $\Phi_{1}$ can be simplified as follows:

(29) minΦ1f1Φ1=2ReTrΦ1HξrHBRU¯UHTRFF¯H−ξtPUHBRUUAUH¯−ξtGHΦ2HU¯DF¯H+ρNRξrHBRHTRHTBH+TrξtPUΦ1HHfleΦ1H¯HH¯+TrξrΦ1HHfleΦ1F¯F¯H+TrξtΦ1HHfraΦ1F¯F¯H+TrρξrNRΦ1HHBRHBRHΦ1HTBHTBHs.t.Φ1ii=1,∀i.

Due to the constant modulus constraint of BIOS, sub-problem (29) remains highly non-convex. Thus, we propose to apply the manifold optimization (MO) algorithm to address this challenge. Similar to the gradient descent method, the MO algorithm aims to find the most rapid descent direction on the manifold which also minimally deviates from it, namely, the Riemann conjugate gradient (RCG). It can be derived that the RCG is the projection of the Euclidean gradient in the tangent space, which is the set of directions that least deviate from the manifold surface. Consequently, the key of MO lies in the derivation of the Euclidean gradient. To obtain the gradient of $f_{1}\left(\Phi_{1}\right)$, we first extract the main diagonal elements of $\Phi_{1}$ containing useful information to form vector $\phi_{1}$, i.e., $\phi_{1}=\mathrm{diag}\left(\Phi_{1}\right)$, and transform the objective function $f_{1}\left(\Phi_{1}\right)$ into $f_{1}\left(\phi_{1}\right)$ as follows:   

(30) f1ϕ1=2ReTrAϕ1H+TrξtPUϕ1HHfleϕ1H¯HH¯+Trξrϕ1HHfleϕ1F¯F¯H+Trξtϕ1HHfraϕ1F¯F¯H+TrρξrNRϕ1HHBRHBRHϕ1HTBHTBH=(a)2Reϕ1Ha+ϕ1HBϕ1,

where A and B are equivalent matrices as

$$\begin{array}{cc} A= & \sqrt{\xi_{r}}H_{\mathrm{BR}}U_{U}A_{U}U_{U}^{H}H_{\mathrm{TR}}F{\bar{F}}^{H}-\sqrt{\xi_{t}P_{U}}H_{\mathrm{BR}}U_{U}A_{U}\bar{H}\\ \; & -\sqrt{\xi_{t}}G^{H}\Phi_{2}^{H}H_{D}A_{D}U_{D}{\bar{F}}^{H}+\frac{\rho }{N_{R}}\sqrt{\xi_{r}}H_{\mathrm{BR}}H_{\mathrm{TR}}H_{\mathrm{TB}}^{H}, \end{array}$$

$$\begin{array}{cc} B= & \xi_{t}P_{U}H_{\mathrm{fle}}⊙{\left({\bar{H}}^{H}\bar{H}\right)}^{T}+\xi_{r}H_{\mathrm{fle}}⊙{\left(\bar{F}{\bar{F}}^{H}\right)}^{T}\\ \; & +\xi_{t}H_{\mathrm{fra}}⊙{\left(\bar{F}{\bar{F}}^{H}\right)}^{T}+\frac{\rho \xi_{r}}{N}\left(H_{\mathrm{BR}}H_{\mathrm{BR}}^{H}\right)⊙{\left(H_{\mathrm{TB}}H_{\mathrm{TB}}^{H}\right)}^{T}, \end{array}$$

and equality (a) holds due to the following properties: $\mathrm{Tr}\left(C^{H}PCQ\right)=c^{H}\left(P⊙Q^{T}\right)c$, $\mathrm{Tr}\left(PC\right)=p^{T}c$ for any matrices P, Q, and diagonal matrix C, with p=diag(P) and c=diag(C). Thus, the Euclidean gradient of $f_{1}\left(\phi_{1}\right)$ can be obtained as

(31) $$\nabla_{\phi_{1}}f_{1}\left(\phi_{1}\right)=a+B\phi_{1}.$$

Thus, the RCG can be derived to facilitate gradient descent with an appropriate step size. Subsequently, the optimized $\phi_{1}$ is projected back onto the manifold surface to ensure the constant modulus constraint. Consequently, the optimal $\phi_{1}$ can be obtained by iteratively descending along the RCG until a predefined stopping criterion is satisfied.

It is important to notice that the term $\mathrm{Tr}\left(\frac{\rho \xi_{r}}{N_{R}}\Phi_{1}^{H}H_{\mathrm{BR}}H_{\mathrm{BR}}^{H}\Phi_{1}H_{\mathrm{TB}}H_{\mathrm{TB}}^{H}\right)$ results from the SI power constraint, while the other terms are associated with the SR. Consequently, the optimization of $\Phi_{1}$ takes into account SRM and SIC simultaneously.

### 3.5. Optimize $\Phi_{2}$ with Fixed F, $\Phi_{1}$, $A_{D}$, $A_{U}$, $U_{D}$, $U_{U}$

Similarly, we optimize $\Phi_{2}$ with other variables fixed in this subsection. By defining equivalent matrices ${\bar{H}}_{\mathrm{BR}}=\sqrt{P_{U}}U_{U}^{H}H_{\mathrm{BR}}^{H}\Phi_{1}G$, ${\bar{H}}_{\mathrm{TB}}=G\Phi_{1}H_{\mathrm{TB}}F$, ${\bar{H}}_{D}=H_{D}U_{D}$, the sub-problem related to $\Phi_{2}$ can be expressed as

(32) minΦ2f2Φ2=−2ξtReTrH¯BRHAUHUH+HDUDADH¯TBHΦ2H+ξtTrΦ2HH¯BRHAUH¯BRΦ2HUHUH+ξtTrΦ2HH¯DADH¯DHΦ2H¯TBH¯TBHs.t.Φ2ii=1,∀i.

Similarly, we can also employ MO to solve (33). Extract the main diagonal elements of $\Phi_{2}$ to form vector $\phi_{2}$; the objective $f_{2}\left(\Phi_{2}\right)$ can be transformed into $f_{2}\left(\phi_{2}\right)$ as follows:

(33) f2Φ2=−2ξtReϕ2Hc+ξtϕ2HDϕ2,

where $\phi_{2}=\mathrm{diag}\left(\Phi_{2}\right)$, and C and D are equivalent matrices as

(34) $$C={\bar{H}}_{\mathrm{BR}}^{H}A_{U}H_{U}^{H}+H_{D}U_{D}A_{D}{\bar{H}}_{\mathrm{TB}}^{H},$$

(35) $$D={\bar{H}}_{\mathrm{BR}}^{H}A_{U}{\bar{H}}_{\mathrm{BR}}⊙{\left(H_{U}H_{U}^{H}\right)}^{T}+{\bar{H}}_{D}A_{D}{\bar{H}}_{D}^{H}⊙{\left({\bar{H}}_{\mathrm{TB}}{\bar{H}}_{\mathrm{TB}}^{H}\right)}^{T}.$$

Thus, the Euclidean gradient of $f_{2}\left(\phi_{2}\right)$ can be obtained as

(36) $$\nabla_{\phi_{2}}f_{2}\left(\phi_{2}\right)=-\sqrt{\xi_{t}}c+\xi_{t}D\phi_{2}.$$

It is worth noting that, despite the similar form, the optimization of $\Phi_{1}$ and $\Phi_{2}$ has different meanings for this system. As indicated in the UL rate expression (12), the SI is tied to $\Phi_{1}$ while not directly related to the optimization of $\Phi_{2}$. Therefore, we can ignore the penalty item related to SIC in (19) during the $\Phi_{2}$ optimization. Furthermore, as the BIOS can independently control the reflection and refraction beams on both sides, the additional freedom enables $\Phi_{2}$ to focus on the beamforming for SRM without affecting the SIC quality.

The proposed SIC-WMMSE algorithm for solving the sub-problem is summarized in Algorithm 1. It is worth noting that the application of the KKT conditions and the MO algorithm enables a reduction in the objective function for each variable within each iteration. When the decrement in the weighted MSE between two iterations is below a predefined threshold, the iteration is terminated. Since the objective function decreases after each step of updating, Algorithm 1 can converge to a locally optimal solution. To analyze its computational complexity, we count the number of complex multiplications of the key steps in one iteration turn. Assuming that $N_{T}=N_{T}=N$, $K_{D}=K_{U}=K$, and M>N>K, the complexity can be summarized as follows: updating $F^{\left(i\right)}$: $O\left(KM^{2}+N^{3}\right)$; updating ${\Phi_{1}}^{\left(i\right)}$: $O\left(M^{3}\right)$; updating ${\Phi_{2}}^{\left(i\right)}$: $O\left(NM^{2}\right)$; updating ${A_{D}}^{\left(i\right)}$: $O\left(KM^{2}\right)$; updating ${A_{U}}^{\left(i\right)}$: $O\left(KM^{2}+MN^{2}\right)$; updating ${U_{D}}^{\left(i\right)}$: $O\left(NK^{2}\right)$; updating ${U_{U}}^{\left(i\right)}$: $O\left(N^{3}\right)$.
**Algorithm 1** Proposed SIC-WMMSE Algorithm1:Randomly initialize $\Phi_{1}^{\left(0\right)}$, $\Phi_{2}^{\left(0\right)}$ and $F^{\left(0\right)}$ and set i=0.2:Calculate $A_{D}^{\left(0\right)}$, $A_{U}^{\left(0\right)}$, $U_{D}^{\left(0\right)}$, and $U_{U}^{\left(0\right)}$ according to (23)–(26).3:**repeat**4:   Update $F^{\left(i\right)}$ according to (28).5:   Update $\Phi_{1}^{\left(i\right)}$, $\Phi_{2}^{\left(i\right)}$ according to (31), (36).6:   Update $U_{D}^{\left(i\right)}$ and $U_{U}^{\left(i\right)}$ according to (24), (26).7:   Update $A_{D}^{\left(i\right)}$, $A_{U}^{\left(i\right)}$ according to (23), (25).8:   i←i+1.9:**until** the stopping condition is satisfied.

## 4. Analysis of BIOS Size

To further guide the practical deployment of BIOS-assisted FD systems, a lower bound of BIOS size required for efficient SIC is derived here for the single-input single-output (SISO) scenario. Specifically, the channels between the ${\mathrm{BS}}_{\mathrm{Tx}}$-${\mathrm{BS}}_{\mathrm{Rx}}$, ${\mathrm{BS}}_{\mathrm{Tx}}$-${\mathrm{IOS}}_{1}$, and ${\mathrm{BS}}_{\mathrm{Rx}}$-${\mathrm{IOS}}_{1}$ are denoted as $h_{\mathrm{TR}}$, $h_{\mathrm{TB}}$, and $h_{\mathrm{BR}}$, respectively.

**Theorem** **1.** *To achieve efficient SIC, the number of elements in one layer of the BIOS must exceed a threshold M that satisfies the following inequality:*

(37) $$\sum_{m=1}^{M}{\left(\frac{1}{d_{m}^{\mathrm{BR}}d_{m}^{\mathrm{TB}}}\right)}^{\frac{3}{2}}\geq \frac{2\sqrt{\pi }}{\sqrt{\xi_{r}}\lambda d_{\mathrm{BB}}d_{\mathrm{TR}}},$$

*where $d_{m}^{\mathrm{BR}},d_{m}^{\mathrm{TB}}$, respectively, denote the distances from the m-th element of the ${IOS}_{1}$ to the ${BS}_{\mathrm{Rx}}$ and ${BS}_{\mathrm{Tx}}$, and $d_{\mathrm{BB}}$ and $d_{\mathrm{TR}}$ denote the distance between BS-BIOS and ${BS}_{\mathrm{Rx}}$-${BS}_{\mathrm{Tx}}$.*

**Proof** **of** **Theorem** **1.** To ensure effective SIC in the SISO scenario, the residual SI should satisfy the following power constraint:

(38) $$∥\left(h_{\mathrm{TR}}+\sqrt{\xi_{r}}h_{\mathrm{BR}}^{H}\Phi_{1}h_{\mathrm{TB}}\right)s_{D}{∥}_{F}^{2}\leq \gamma .$$

Notably, for effective SIC, the threshold γ should be minimized, and the power constraint (38) should be universally valid for any $s_{D}$. This requires that the residual interference approaches zero, which can be expressed as

(39) $$\left(h_{\mathrm{TR}}+\sqrt{\xi_{r}}h_{\mathrm{BR}}^{H}\Phi_{1}h_{\mathrm{TB}}\right)\Rightarrow 0.$$

To clarify, the necessary condition for efficient SIC is that the modulus of the cascaded channel $\sqrt{\xi_{r}}h_{\mathrm{BR}}^{H}\Phi_{1}h_{\mathrm{TB}}$ should exceed that of the SI channel $h_{\mathrm{TR}}$, which can be formulated as follows:

(40) $${\left|\sqrt{\xi_{r}}h_{\mathrm{BR}}^{H}\Phi h_{\mathrm{TB}}\right|}^{2}\geq {\left|h_{\mathrm{TR}}\right|}^{2}.$$

We define g(M) as the maximum gain of the cascade channel generated by BIOS with *M* scattering elements in one layer, which is given by

(41) $$\begin{array}{c} g\left(M\right)=\max_{\Phi }{\left|\xi_{r}h_{\mathrm{BR}}^{H}\Phi h_{\mathrm{TB}}\right|}^{2}=\max_{\phi }\xi_{r}{\left|\sum_{m=1}^{M}{\left[h_{\mathrm{BR}}^{H}\right]}_{m}{\left[\phi \right]}_{m}{\left[h_{\mathrm{TB}}\right]}_{m}\right|}^{2} \end{array}$$

It can be observed that maximizing the cascade channel gain occurs when the phase shift element ${\left[\phi \right]}_{m}$ is aligned in phase with ${\left[h_{\mathrm{BR}}^{H}\right]}_{m}{\left[h_{\mathrm{TB}}\right]}_{m}$. Thus, by setting ${\left[\phi_{1}\right]}_{m}=\frac{{\left[h_{\mathrm{BR}}\right]}_{m}{\left[h_{\mathrm{TB}}^{*}\right]}_{m}}{\left|{\left[h_{\mathrm{BR}}^{*}\right]}_{m}{\left[h_{\mathrm{TB}}\right]}_{m}\right|}$, g(M) has an evident closed-form solution as

(42) $$\begin{array}{c} g\left(M\right)=\xi_{r}{∥h_{\mathrm{BR}}\circ h_{\mathrm{TB}}∥}_{1}^{2}=\xi_{r}{\left(\sum_{m=1}^{M}\left|{\left[h_{\mathrm{BR}}^{*}\right]}_{m}{\left[h_{\mathrm{TB}}\right]}_{m}\right|\right)}^{2}. \end{array}$$

By substituting the channel settings, we have

(43) $$g\left(M\right)=\frac{\lambda^{2}d_{\mathrm{BB}}}{8\pi }\sum_{m=1}^{M}{\left(\frac{1}{d_{m}^{\mathrm{BR}}d_{m}^{\mathrm{TB}}}\right)}^{\frac{3}{2}}.$$

(44) $$\left|h_{\mathrm{TR}}\right|=\frac{\lambda }{4\sqrt{\pi }d_{\mathrm{TR}}}.$$

According to the derivations mentioned above, the lower bound of the element number can be derived through the inequality constraint

(45) $$\frac{\lambda^{2}d_{\mathrm{BB}}}{8\pi }\sum_{m=1}^{M}{\left(\frac{1}{d_{m}^{\mathrm{BR}}d_{m}^{\mathrm{TB}}}\right)}^{\frac{3}{2}}\geq \frac{\lambda }{4\sqrt{\pi }d_{\mathrm{TR}}}.$$

The proof is thus completed. □

Theorem 1 provides a lower bound of BIOS size required for SIC, which is of practical importance in the design of FD systems. As will be shown in Section 5, the derived lower bound is rather close to the required BIOS size in the simulation.

## 5. Simulation Results

This section presents numerical results to illustrate the performance evaluation of the proposed BIOS-assisted FD system. For comparison, an IOS-assisted FD system is employed as a benchmark, which is characterized by identical phase matrices for reflecting and refracting with $\xi_{r}=\xi_{t}=1/2$. Throughout simulations, the centers of the BS and BIOS are set at the origin of a three-dimensional coordinate system. The distance between two neighboring IOSs of the BIOS is set as $d_{\mathrm{II}}=0.05$ m. The DL and UL UEs are randomly distributed within two respective circles of 3m radius centered at (20,−10,0) and (10,10,0). Considering the NLoS paths, $h_{D,k}$ and $h_{U,l}$ are modeled as Rician channels with a Rician factor κ=4. Moreover, $\alpha_{0}=10^{-4}$ is the pass loss of the reference distance $d_{0}=1$ m with the path loss exponent β=2.2. Furthermore, λ=0.05 m and $\sigma_{U}^{2}=\sigma_{D}^{2}=-80$ dBm.

**Figure 2:**
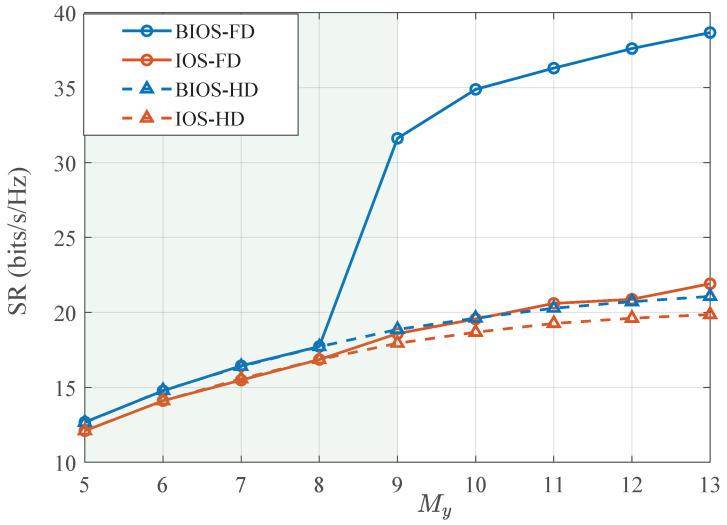
SR performance of BIOS/IOS-assisted systems with different $M_{y}$ ($P_{s}$ = 10 dBm, $P_{u}$ = 7 dBm, $d_{\mathrm{TR}}=3\lambda$, $d_{\mathrm{BB}}=1.5\lambda$).

Figure 2 shows the SR performance as a function of the BIOS/IOS size for different designs in a multi-user MISO system with $N_{T}=N_{R}=3$ and $K_{D}=K_{U}=2$. Specifically, the solid lines illustrate the SR of FD systems, and the dashed lines indicate the rate of HD systems, with blue for BIOS assistance and red for IOS assistance, respectively. Meanwhile, as the BIOS may be too small to enable effective SIC, we switch the system from the FD mode to the HD one with only the DL transmission when the SIC constraint cannot be satisfied, which is indicated by the shaded area. It is worth noting that as the channels are randomly generated, the shaded area means that in most of the simulation realizations, the system has to switch to the HD mode as the size of the BIOS/IOS is too small to achieve the SIC threshold. It can be observed that for all systems, the data rate monotonically increases with $M_{y}$ for both BIOS and IOS due to the enhanced beamforming gains. For FD systems, as $M_{y}$ increases, the SR of BIOS-assisted systems significantly outperforms that of HD systems. However, the IOS-assisted FD system demonstrates only a slight enhancement over HD systems. This is because the IOS beams for SIC and SRM are tightly coupled, limiting the system’s efficiency. Specifically, if the IOS is designed to cancel the SI signal on the reflecting side, it cannot be efficiently optimized for SRM on the refracting side. Conversely, the BIOS-assisted FD system can provide flexible beamforming on both sides, thereby enhancing communication performance and achieving SIC simultaneously.

**Figure 3:**
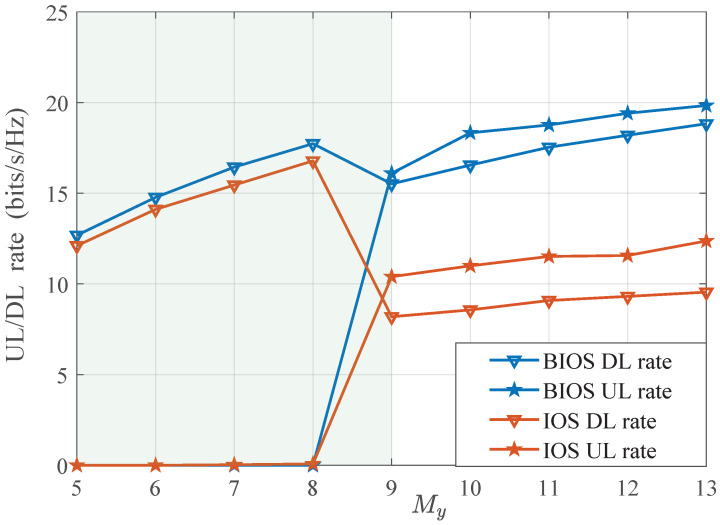
DL/UL rate performance of BIOS/IOS-assisted systems with different $M_{y}$.

To further investigate the beamforming performance of the BIOS and IOS in FD systems, Figure 3 shows the detailed UL and DL rates of the BIOS/IOS-assisted system with different *M*. As can be observed, the system switches from the FD mode to the HD mode with $M_{y}<9$. In this range, the data rate, i.e., the DL rate, consistently increases with *M*. This is because the expansion of the array scale provides higher degrees of freedom, thus enhancing the beamforming gain. However, when $M_{y}$ changes from 8 to 9, the DL rate unexpectedly decreases while the UL rate substantially increases. This is because the power of the cascaded signal generated by the BIOS/IOS is strong enough to facilitate effective SIC, thus leading to a trade-off between the SRM and SIC for beamforming. It is worth noting that, compared with the IOS-assisted system, the DL rate in the BIOS-assisted system is less affected due to the independent beamforming on both sides. Consequently, the SR performance of the BIOS-assisted FD system significantly surpasses that of the IOS-assisted system.

**Figure 4:**
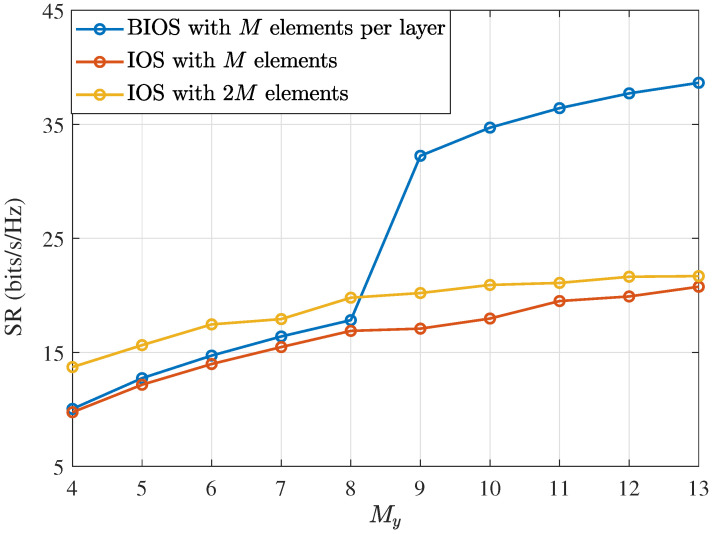
SR performance of BIOS/IOS-assisted systems with different $M_{y}$ ($P_{s}$ = 10 dBm, $P_{u}$ = 7 dBm, $d_{\mathrm{TR}}=3\lambda$, $d_{\mathrm{BB}}=1.5\lambda$).

Furthermore, Figure 4 shows the performance comparison between the proposed BIOS and an IOS with the same scattering elements. As the element spacing is kept unchanged, the IOS with 2M elements has a larger aperture size, it can be observed that the IOS with 2M elements slightly outperforms the IOS with *M* elements due to the additional beamforming gain from the increase in the element number and the aperture size. Similarly, it outperforms the BIOS when $M_{y}$ is small for the same reasons. However, with the increase in $M_{y}$, the performance of the IOS with 2M elements remains significantly inferior to that of the BIOS due to the coupling between the reflected and refracted beams.

**Figure 5:**
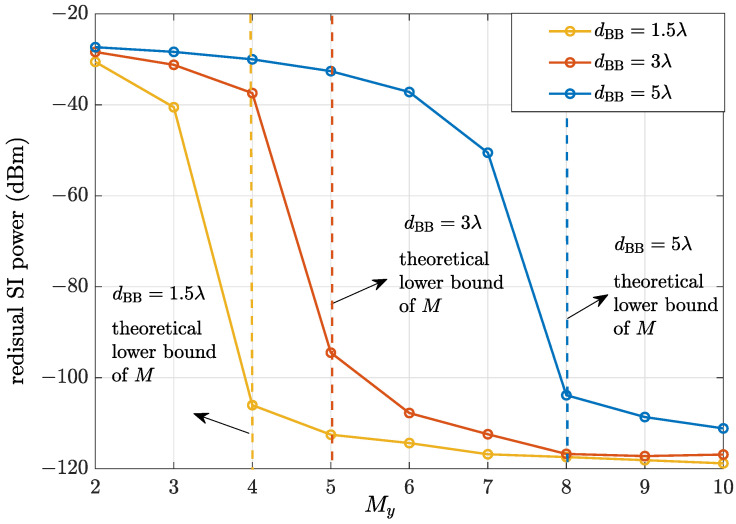
Residual SI power of BIOS-assisted FD systems with different $M_{y}$.

Finally, to visualize the SIC capability of the BIOS and verify the theoretical lower bound discussed in Section 4, Figure 5 shows the residual SI power as a function of $M_{y}$ in SISO FD systems with different $d_{\mathrm{BB}}$. Specifically, the solid line represents the residual SI power, and the dashed line of the same color represents the theoretical lower bound of *M* for efficient SIC in the system with the same $d_{\mathrm{BB}}$. It can be observed that the residual SI power with the proposed scheme monotonically decreases as *M* increases due to the beamforming gain. Meanwhile, despite the approximation, the theoretical lower bound calculated according to (37) is consistent with the simulation result.

## 6. Conclusions

We proposed a novel BIOS-assisted FD system for simultaneous SIC and SRM, and formulated the beamforming optimization problem aiming at maximizing the SR with the SIC requirement constraint. The BS precoder and the BIOS phase shifts were jointly optimized by the proposed SIC-WMMSE approach. Furthermore, a theoretical lower bound of BIOS size required for efficient SIC was derived and proved to be very tight through simulation. The simulation results also showed that the BIOS-assisted FD system significantly outperforms the conventional IOS-assisted system, as the BIOS can flexibly control the beamforming for SIC and SRM.

## Data Availability

Data are contained within the article.

## Appendix A

**Theorem** **A1.**
*For the problem of SRM for both DL and UL, it can be transformed into a WMMSE problem by introducing auxiliary variables $a_{D,k}$ and $a_{U,l}$, i.e., weighting coefficients, which can be reformulated as follows:*

(A1)
$$\arg\max_{\Phi_{1},\Phi_{2},F}R_{D,k}=\arg\min_{\Phi_{1},\Phi_{2},F}\left(a_{D,k}e_{D,k}-\log_{2}\left|a_{D,k}\right|\right),\;\forall k,$$

(A2)
$$\arg\max_{\Phi_{1},\Phi_{2},F}R_{U,l}=\arg\min_{\Phi_{1},\Phi_{2},F}\left(a_{U,l}e_{U,l}-\log_{2}\left|a_{U,l}\right|\right),\;\forall l.$$

**Proof** **of** **Theorem** **A1.** In the DL scenario, considering the independence between UEs, we take the *k*-th DL UE as an example to prove the equivalence between SRM and WMMSE. The proof is to demonstrate that maximizing the rate of the *k*-th DL UE is equivalent to solving the following optimization problem:

(A3) $$\min_{\Phi_{1},\Phi_{2},F}\;g_{D,k}=a_{D,k}e_{D,k}-\log_{2}\left|a_{D,k}\right|.$$

It can be observed that when variables $\Phi_{1},\Phi_{2}$, and F are fixed, the optimization problem (A3) becomes convex with respect to $A_{D,k}$, $u_{D,k}$. Thus, the optimal $A_{D,k}$ can be directly obtained through the first-order conditions

(A4) $$\nabla_{a_{D,k}}\left(g_{D,k}\right)=0\Rightarrow a_{D,k}^{★}=e_{D,k}^{-1}.$$

Similarly, the optimal $u_{D,k}$ can be derived as

(A5) ∇uD,k*gD,k=aD,k∂uD,k*uD,kh^D,kHFFHh^D,k−2ReuD,k*h^D,kHfk+σD,k2uD,k*uD,k+1∂uD,k*=aD,kuD,kh^D,kHFFHh^D,k−h^D,kHfk+σD,k2uD,k,

(A6) $$\nabla_{u_{D,k^{*}}}\left(g_{D,k}\right)=0\Rightarrow {u_{D,k}}^{★}={\left({\hat{h}}_{D,k}^{H}FF^{H}{\hat{h}}_{D,k}+\sigma_{D,k}^{2}\right)}^{-1}{\hat{h}}_{D,k}^{H}f_{k}.$$

By substituting ${u_{D,k}}^{★}$ into $e_{D,k}$, the minimized MSE can be derived as follows:

(A7) $$\begin{array}{cc} e_{D,k}^{\mathrm{MMSE}} & =1-{\hat{h}}_{D,k}^{H}f_{k}f_{k}^{H}{\hat{h}}_{D,k}{\left({\hat{h}}_{D,k}^{H}FF^{H}{\hat{h}}_{D,k}+\sigma_{D}^{2}\right)}^{-1}\\ \; & =\frac{\sum_{i=k,i=1}^{K_{D}}{\hat{h}}_{D,k}^{H}f_{i}f_{i}^{H}{\hat{h}}_{D,k}+\sigma_{D}^{2}}{{\hat{h}}_{D,k}^{H}FF^{H}{\hat{h}}_{D,k}+\sigma_{D}^{2}}. \end{array}$$

According to (A4), the optimal $a_{D,k}^{★}$ can be rewritten as

(A8) $${a_{D,k}}^{★}=e_{D,k}^{\mathrm{MMSE}-1}=1+\frac{{\hat{h}}_{D,k}^{H}f_{k}f_{k}^{H}{\hat{h}}_{D,k}}{\sum_{i\neq k,i=1}^{K_{D}}{\hat{h}}_{D,k}^{H}f_{i}f_{i}^{H}{\hat{h}}_{D,k}+\sigma_{D,k}^{2}},$$

By taking (A8) back to $g_{D,k}$, we have

(A9) $$g_{D,k}=1-\log\left|a_{D,k}\right|=1-\log\left(1+\frac{{\hat{h}}_{D,k}^{H}f_{k}f_{k}^{H}{\hat{h}}_{D,k}}{\sum_{i=k,j=1}^{K_{D}}{\hat{h}}_{D,f_{i}}^{f}f_{i}^{H}{\hat{h}}_{D,k}+\sigma_{D,k}^{2}}\right).$$

Comparing (11) with (A9), we can find that the logarithmic term in (A9) matches the expression of the achievable rate. Consequently, the optimal solution ${{\Phi_{1}}^{★},{\Phi_{2}}^{★},F}^{★}$ that maximizes the rate in (11) is also the one that minimizes the WMMSE as described in (A9). Similarly, we take the *l*-th UL UE as an example to prove the equivalence between SRM and WMMSE in the UL scenario. This equivalence is shown by transforming the rate maximization problem of the *l*-th UL UE into the following minimization problem

(A10) $$\min_{\Phi_{1},\Phi_{2},F}\;g_{U,l}=a_{U,l}e_{U,l}-\log_{2}\left|a_{U,l}\right|.$$

With $\Phi_{1},\Phi_{2},F$ fixed, the optimal $a_{U,l}$, $u_{U,l}$ can be derived as

(A11) $$\nabla_{a_{U,1}^{★}}\left(g_{U,l}\right)=0\Rightarrow a_{U,l}^{★}=e_{U,l}^{-1},$$

(A12) $$\begin{array}{cc} \nabla_{u_{U,l}}{(}_{U,l}) & =0\\ \Rightarrow {u_{U,l}}^{★} & =\sqrt{P_{U}}{\left(P_{U}{\hat{H}}_{U}{\hat{H}}_{U}^{H}+H_{\mathrm{SI}}FF^{H}H_{\mathrm{SI}}^{H}+\sigma_{U}^{2}I\right)}^{-1}{\hat{h}}_{U,l}, \end{array}$$

where

(A13) ∇uU,gU,l=∂aU,leU,l∂uU,lH=aU,l∂uU,lHPUH^UH^UH+HSIFFHHSIH+σU2IuU,l−2PUReuU,lHh^U,l+1∂uU,lH=aU,lPUH^UH^UH+HSIFFHHSIH+σU2IuU,l−PUh^U,l.

By substituting ${u_{U,l}}^{★}$ into (16), we have

(A14) eU,lMMSE=uU,lHPUH^UH^UH+HSIFFHHSIH+σU2IuU,l−2PUReuU,lHh^U,l+1=1−PUh^U,lHPUH^UH^UH+HSIFFHHSIH+σU2I−1h^U,l.

Thus, according to (A11), $a_{U,l}^{★}$ can be rewritten as

(A15) $$\begin{array}{cc} {a_{U,l}}^{★} & =e_{U,l}^{\mathrm{NMSE}-1}\\ \; & \overset{\left(b\right)}{=}1+P_{U}{\hat{h}}_{U,l}{\left(P_{U}\sum_{i=1,i\neq l}^{K_{U}}{\hat{h}}_{U,i}{\hat{h}}_{U,i}^{H}+H_{\mathrm{SI}}FF^{H}H_{\mathrm{SI}}^{H}+\sigma_{U}^{2}I\right)}^{-1}{\hat{h}}_{U,l}^{H}, \end{array}$$

where equality (b) holds due to the Woodbury matrix identity ${\left(A+\mathrm{UCV}\right)}^{-1}=A^{-1}-A^{-1}U{\left(C^{-1}+{\mathrm{VA}}^{-1}U\right)}^{-1}{\mathrm{VA}}^{-1}$. By taking (A11) back to $g_{U,l}$, we have

(A16) $$\begin{array}{cc} g_{U,l} & =1-\log\left|e_{U,l}^{\mathrm{MMSE}-1}\right|\\ \; & =1-\log\left|1+P_{U}{\hat{h}}_{U,l}^{H}{\left(P_{U}\sum_{i=1,i\neq l}^{K_{U}}{\hat{h}}_{U,i}{\hat{h}}_{U,i}^{H}+H_{\mathrm{SI}}FF^{H}H_{\mathrm{SI}}^{H}+\sigma_{U}^{2}I\right)}^{-1}{\hat{h}}_{U,l}\right|\\ \; & \overset{\left(c\right)}{=}1-\log\left|I+\frac{P_{U}{\hat{h}}_{U,l}{\hat{h}}_{U,l}^{H}}{P_{U}\sum_{i=1,i\neq l}^{K_{U}}{\hat{h}}_{U,i}{\hat{h}}_{U,i}^{H}+H_{\mathrm{SI}}FF^{H}H_{\mathrm{SI}}^{H}+\sigma_{U}^{2}I}\right|, \end{array}$$

where equality (c) holds due to $\det\left(I+uv^{H}\right)=1+v^{H}u$. Similarly, comparing (12) with (A16), we can find that the logarithmic term in (A16) directly corresponds to the achievable rate. Therefore, the optimal solution ${{\Phi_{1}}^{*},{\Phi_{2}}^{*},F}^{*}$ that maximizes the rate in (12) is also the one that minimizes the WMMSE(A10). The proof is thus complete. □
